# Bioinformatics combined with machine learning for the identification of malignant transformation markers in colorectal polyps

**DOI:** 10.3389/fmolb.2026.1785464

**Published:** 2026-03-24

**Authors:** Tao He, Caixia Liang, Kui Li, Dagang Li

**Affiliations:** 1 School of Computer Science and Engineering, Macau University of Science and Technology, Taipa, Macau, China; 2 Party and Government Office, Nantong First People’s Hospital, Affiliated Hospital 2 of Nantong University, Nantong, Jiangsu, China; 3 Medical Research Center, Nantong First People’s Hospital, Affiliated Hospital 2 of Nantong University, Nantong, Jiangsu, China; 4 Information Department, Nantong First People’s Hospital, Affiliated Hospital 2 of Nantong University, Nantong, Jiangsu, China

**Keywords:** bioinformatics, colorectal cancer (CRC), machine learning, malignant transformation markers, polyps

## Abstract

**Background:**

Colorectal polyps, as crucial precancerous lesions of colorectal cancer (CRC), have incompletely clarified origin and evolutionary mechanisms, which restrict the early prevention and control of CRC. This study aimed to screen core genes regulating colorectal tumorigenesis and construct a reliable diagnostic model for CRC.

**Methods:**

The edgeR package and weighted gene co-expression network analysis (WGCNA) were first used to analyze the GSE209741 dataset to identify differentially expressed genes (DEGs) and module genes, followed by functional enrichment analysis to reveal core biological pathways and functions. Combined with the GSE161277 single-cell RNA sequencing dataset, 57 epithelial cell-specific regulatory molecules were screened. Based on the TCGA-COADREAD cohort, feature genes were selected by the combined application of the Boruta algorithm, LASSO regression and XGBoost model. Finally, a ridge regression diagnostic model was established using six core genes (EIF2S3, GTF3A, HMGA1, HSP90AB1, PABPC1, S100A11), and its performance was verified in the internal validation set and the external independent cohort GSE41258. Meanwhile, the UALCAN database was used to validate the protein expression levels of core genes in tumor tissues, survival analysis was performed to explore their correlation with CRC prognosis, and qRT-PCR was applied to verify the mRNA expression differences of the six core genes between CRC cell lines (SW480, HCT116) and the normal colorectal epithelial cell line NCM460.

**Results:**

The diagnostic model exhibited excellent diagnostic efficacy in both internal and external datasets. The UALCAN database confirmed that the protein expression of the six genes was significantly upregulated in CRC tissues. Survival analysis revealed that high expression of EIF2S3 and S100A11 was associated with poor prognosis in CRC patients. qRT-PCR further verified that the mRNA expression levels of the six core genes were significantly elevated in CRC cell lines.

**Conclusion:**

This study identified six key genes regulating colorectal tumorigenesis and constructed a high-performance diagnostic model. These findings provide novel insights into the molecular mechanisms underlying the initiation and progression of CRC, and offer potential biomarkers and therapeutic targets for the clinical diagnosis and treatment of CRC.

## Introduction

Colorectal cancer (CRC) is one of the most common malignant tumors worldwide, ranking as the third most prevalent and second most lethal malignancy in 2022 according to the latest GLOBOCAN data. The burden of CRC is projected to increase to 3.2 million new cases and 1.6 million deaths by 2040 (Bray et al., 2024; Bray et al., 2018). Early and accurate diagnosis is crucial for reducing the morbidity and mortality of CRC patients as well as improving their survival rates. Current diagnostic methods include stool-based tests, blood-based tests, direct visualization, and imaging examinations. However, the current early diagnostic methods have limitations (Liu and Zhang, 2024; Tajbakhsh et al., 2014). In addition, the understanding of the underlying molecular mechanisms that influence CRC progression is not yet complete, which hinders the development of more precise and effective treatment strategies.

CRC usually develops from a benign growth known as a colorectal polyp, which can develop into an invasive cancer. Colorectal polyps from both an adenoma and a serrated polyp have a significant risk of developing into CRC (Łukaszewicz-Zając and Mroczko, 2021; Valle and Monahan, 2024). Endoscopic detection and removal of adenomas can significantly reduce the incidence and mortality of CRC (Zauber et al., 2012; Levin et al., 2008). In addition, the probability of a polyp progressing to cancer depends not only on its histological classification but also on its genetic profile (Chen et al., 2021; Kim et al., 2011). Martínez-Roca A et al. shows that somatic mutations in KRAS in at least one polyp at baseline colonoscopy are an independent risk factor for the development of advanced metachronous adenomas (Martínez-Roca et al., 2024). Zheng X et al. performed single-cell RNA sequencing on cells from patient-matched tissue samples, including blood, normal tissue, para-cancer, polyp, and colorectal cancer, clarified the cellular characteristics at each stage, identified BMX and HCK as potential drivers of adenoma initiation, and found that their overexpression promotes cell proliferation and the formation of polyp-like morphological structures (Zhu et al., 2024). Therefore, understanding the molecular mechanisms of the transformation from polyps to CRC will provide clues for the early interception and prevention of CRC progression. However, the driving events of tumorigenesis in precursor cell populations and stage-specific molecules remain unclear at present, which urgently require further exploration.

High-throughput sequencing technology has demonstrated significant application value in the field of disease diagnosis and treatment due to its characteristics of efficiently and accurately analyzing genetic information (Zhu et al., 2024; Byrne et al., 2024). Single-cell RNA sequencing (scRNA-seq) has enhanced our understanding of cellular heterogeneity, novel phenotypic states and provided revolutionary insights for a wide range of pathological studies (Wälchli et al., 2024; Tirosh et al., 2016). The scRNA-seq technique has been extensively employed in CRC research. To map the regulatory and transcriptomic changes that occur along the phenotypic continuum from healthy colon to invasive cancer, Becker WR et al. analyzed the cell types in sporadic polyps and those of FAP patients using snRNA-seq and ATAC-seq assay to identify the cell types and transcriptional regulatory programs within these polyps (Becker et al., 2022). Sheng J et al. performed scRNA-seq on 51,819 single cells from 10 clinical samples, revealing the molecular signatures of 10 major cell types and 39 subtypes, presenting a dynamic stage-specific molecular landscape, and identifying the crucial roles of ligand-receptor genes, transcription factors, and other factors in colorectal carcinogenesis (Sheng et al., 2025). However, current research on the transformation of healthy colon to precancerous adenomas to CRC remains limited. scRNA-seq combined with multi-omics and experimental validation is crucial for identifying stage-specific driver genes and actionable therapeutic windows.

The rapid development of omics technologies has provided an enormous volume of biological data for CRC research. While such data contain critical clues for exploring the pathogenesis and molecular biomarkers of CRC, their high-dimensionality and strong correlation pose great challenges to traditional analytical methods. In contrast, machine learning (ML) techniques have exhibited remarkable advantages in overcoming this bottleneck by virtue of their outstanding capabilities in complex data processing and pattern recognition (He et al., 2020; Hou et al., 2025; Liu et al., 2022). For instance, Liu et al. integrated multi-dimensional bioinformatics analyses with machine learning algorithms to systematically investigate the correlation patterns between immune-related long non-coding RNA (lncRNA) profiles and the prognosis, recurrence risk, and degree of drug benefit in CRC patients (Liu et al., 2022). Wei et al. innovatively combined traditional statistical tests with machine learning techniques, which effectively improved the stability and interpretability of molecular biomarker identification in CRC (Wei et al., 2023). Yang et al. first used machine learning approaches to confirm that the microbial composition in CRC tumor tissues is closely associated with patient survival and prognosis (Yang et al., 2022).

This study aims to elucidate the malignant evolution mechanisms of CRC and identify therapeutic targets with potential clinical translational value. A systematic strategy integrating multi-dataset analysis and machine learning was adopted as follows: first, DEGs were screened from the GSE209741 dataset, and WGCNA was performed to identify core gene modules closely correlated with disease progression. Subsequently, the single-cell RNA sequencing dataset GSE161277 was utilized to map the expression localization of core genes across distinct cellular subpopulations, while simultaneously characterizing their dynamic expression patterns throughout the malignant transformation cascade spanning normal tissues, precancerous lesions, and cancerous tissues—thus providing single-cell resolution evidence to decipher the intrinsic links between gene function and CRC pathogenesis. On this basis, machine learning algorithms were applied to filter core feature genes; a ridge regression-based diagnostic model was then established and comprehensively validated, ultimately pinpointing key biomarkers that mediate the malignant transformation of colorectal polyps into CRC. Taken together, these findings are anticipated to furnish critical theoretical and experimental support for the early warning, precise intervention, and development of targeted therapeutics against CRC.

## Materials and methods

### Data collection

**FIGURE 1:**
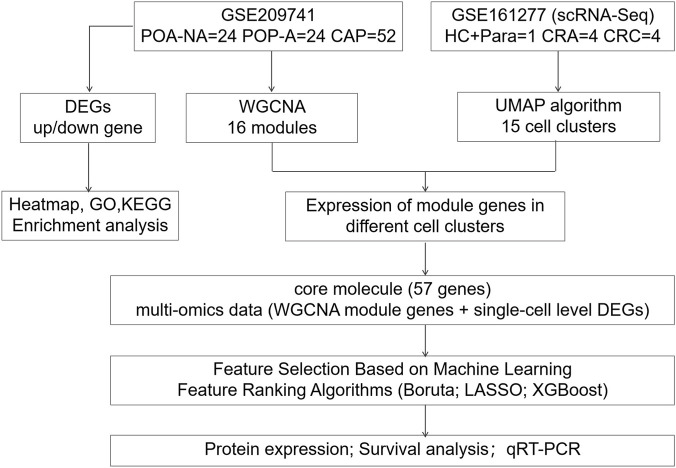
Flowchart of this research. POP-NA: non-aggressive polyps; POP-A: aggressive polyps; CAP: cancer adjacent polyps; HC-Para: paracancerous tissues; CRA: adenomatous tissues; CRC: cancerous tissues; scRNA-seq: single-cell RNA sequencing; DEGs: differentially expressed genes; WGCNA: weighted gene co-expression network analysis; UMAP: uniform manifold approximation and projection; GO: gene ontology; KEGG: Kyoto Encyclopedia of Genes and Genomes.

Through systematic screening of the GEO database (Gene Expression Omnibus, https://www.ncbi.nlm.nih.gov/geo/), the search keywords were set as “colorectal polyps”, “array”, and “*Homo sapiens*”. The inclusion criteria for datasets were established as follows: (1) each sample group must include at least 3 independent patients; (2) the annotation platform (GPL) must provide complete information on Gene Symbols and Entrez IDs. Three bulk RNA-seq datasets were ultimately sincluded in this study: TCGA-COADREAD, GSE209741, and GSE41258. All three datasets include substantial sample sizes and were generated using classical profiling platforms—either the Affymetrix Human Genome U133A Array or RNA sequencing—thereby ensuring high data quality and reliability. Specifically, TCGA-COADREAD provides primary tumor and normal tissue samples from The Cancer Genome Atlas, with corresponding clinical data downloaded from UCSC Xena (https://xena.ucsc.edu/). GSE209741 includes sample data of cancer-free polyps (non-aggressive, aggressive) and cancer-adjacent polyps from 100 patients, covering whole-genome and RNA sequencing results, with over 300 tissue samples. GSE41258 is a well-characterized colorectal cancer dataset generated using the Affymetrix platform, offering robust gene expression profiles. In addition, one single-cell RNA sequencing dataset, GSE161277, was included. It is a patient-matched multi-tissue scRNA-seq dataset, comprising samples from paracancerous tissues, adenomatous tissues, and cancerous tissues (Zheng et al., 2022). The clinical information for each dataset is provided in Supplementary Table S1. The overall design workflow of this study is shown in Figure 1.

### Differentially expressed genes (DEGs) analysis

The DEG analysis was performed on the RNA-seq data using R edgeR package(v3.32.1). The selection criteria for DEGs were set as |logFC| ≥ log2(1.5) and FDR<0.05. Heatmap analysis and enrichment analysis were conducted on the screened differential genes.

### Weighted gene co-expression network analysis (WGCNA) network construction and module identification

WGCNA is performed to identify gene modules with high synergistic effects, summarize the interconnections between modules as well as the associations between modules and phenotypes, and screen out candidate biomarkers or therapeutic targets (Chen et al., 2025; Zhao et al., 2025). In this study, WGCNA was constructed by the R package “WGCNA” to identify the modules associated with polyps and colon cancer. The specific steps are as follows: First, samples were grouped to check for obvious outliers. Subsequently, an appropriate soft-thresholding power β = 16 was selected according to the criteria of a scale-free network. Then, a hierarchical clustering dendrogram was constructed, and genes with similar expression patterns were classified into different modules. Finally, by calculating the correlation between Module Eigengenes (ME) and clinical features, suitable gene modules were screened out for subsequent research. By calculating the Pearson correlation coefficients between module eigengenes and clinical traits, modules with |correlation coefficient| > 0.3 and P < 0.05 were retained as key modules significantly associated with clinical phenotypes for subsequent analysis.

### scRNA-seq data analysis

The public scRNA-seq datasets were retrieved from the database (GSE161277) (Zheng et al., 2022). For quality control, cells were kept based on the following criteria: number of detected genes (nFeature_RNA) between 200 and 6,000, total UMI counts (nCount_RNA) between 500 and 50,000, mitochondrial gene percentage (percent.mt) below 25%, and log10(genes per UMI) > 0.8. Genes expressed in fewer than three cells were also excluded. After preliminary quality control, the data were first subjected to normalization and scaling; subsequently, anchors were identified between different cell population identities, and these anchors were applied to the “IntegrateData” method to reduce batch effects among samples from different individuals. During the data visualization phase, the uniform manifold approximation and projection (UMAP) method was used for further dimensionality reduction. To cluster single cells based on their gene expression profiles, this study adopted the unsupervised graph-based Louvain clustering algorithm, and canonical marker genes were used to complete cell type annotation. All the above analysis operations were implemented using the Seurat (4.0) R package. The gene expression patterns in the single-cell datasets were visualized using scattered plots in the two-dimensional UMAP dimensions.

### Pseudotime analysis

Single-cell trajectory analysis was conducted *via* the Monocle v2.14.0 R package, which uses machine learning to decode temporal gene expression patterns in cell populations during dynamic biological processes. It accurately captures continuous cell trajectories from initial to terminal states, providing a quantitative framework to explore cell fate transition mechanisms (Qiu et al., 2017). The workflow was as follows: (1) Data Preparation: CellDataSet (CDS) objects were built with default parameters for each cell type’s single-cell transcriptomic data, integrating gene expression profiles to support subsequent analysis. (2) Ordering Gene Selection: Differential expression analysis compared cells at the biological process’s initial and terminal states to screen ordering genes. Exon sequencing validated the normal tissue-adenoma-carcinoma evolutionary relationship to ensure these genes reflected cell state temporal transitions. (3) Dimensionality reduction and trajectory modeling: The reversed graph embedding algorithm (based on ordering genes) projected high-dimensional data into low-dimensional space, constructed a smooth tree-like manifold, and generated a trajectory model capturing cell dynamic progression from original to differentiation states. As a complementary approach to Monocle2, fibroblast trajectory analysis was performed using Monocle3 V1.4.26 R package with defalut parameter. Based on prior knowledge, normal epithelials were selected as the trajectory root, and the inferred differentiation paths were visualized on UMAP.

### Feature selection

RNA-seq transcriptome data and corresponding clinical information for the TCGA-COADREAD cohort were obtained from The Cancer Genome Atlas (TCGA) database, retaining only samples annotated as primary tumor and solid tissue normal. This curated cohort was then randomly partitioned into a training set and an internal test set at a 7:3 ratio. To identify robust diagnostic signatures, a stringent feature selection process was applied exclusively to the training set, starting from a pre-defined panel of 57 candidate genes. Feature selection employed a three-step approach combining Boruta, LASSO, and XGBoost to enhance robustness. First, Boruta (v8.0.0) was run with 500 iterations (maxRuns = 500) and pValue = 0.01; tentative attributes were resolved using TentativeRoughFix(), retaining only those confirmed as important. Second, LASSO logistic regression (glmnet v4.1.8) with 10-fold cross-validation selected features with non-zero coefficients at the λ that maximized AUC (lambda.min). Third, XGBoost (binary:logistic) was trained with five-fold cross-validation and early stopping (max 200 rounds, early_stopping_rounds = 20). Features were ranked by Gain, and the top contributors accounting for 95% cumulative Gain were kept. All steps used a fixed random seed (set.seed(123)) for reproducibility. The genes commonly selected by all three methods constituted the final feature set. Subsequently, a Ridge Regression model was constructed using the expression profiles of these selected genes within the training set to predict diagnostic outcomes. The model’s performance was systematically evaluated; its discriminative ability was first assessed on the held-out TCGA test set using metrics including the Area Under the Receiver Operating Characteristic Curve (AUC-ROC), accuracy, sensitivity, and specificity. To further validate the model’s robustness and generalizability, it was independently tested on an external validation cohort, GSE41258.

### Cell culture

Human colorectal cancer cell lines (HCT116, LOVO, and RKO) were purchased from the ATCC, and the normal human colon mucosal epithelial cells (NCM460) was obtained from Yuchun Biotechnology Co., Ltd. (Shanghai, China). HCT116, RKO cells were cultured in 1,640 medium supplemented with 10% fetal bovine serum (Gibico, 10,091,148, United States of America) and 100 U/mL penicillin/streptomycin at 37 °C with 5% CO2. LOVO and NCM460 cells were maintained in DMEM medium supplemented with 10% FBS and 100 U/mL penicillin/streptomycin. Upon reaching approximately 90% confluence, all cells were harvested for subsequent experiments.

### RNA extraction and qRT-PCR

For each sample, total RNA was extracted using TRIzol reagent (Invitrogen) according to the manufacturer’s instructions. After extraction, the concentration and purity of total RNA were quantified using a NanoDrop 2000 spectrophotometer (Thermo Fisher Scientific, Waltham, MA, United States of America).

Total RNA (1 μg) was reverse-transcribed to cDNA using a PrimeScript RT reagent kit (TaKaRa, RR036A, Japan). The cDNA (3-fold dilution) was used to perform qRT-PCR in triplicate in a 20 μL mixture by using an SYBR Green kit (Beyotime, D7262, China) with specific primers on CFX Connect (Bio-Rad, QX200, United States of America). The mRNA level was analysed by using the 2^−ΔΔCT^ method. β-actin was used as a reference gene. All premier sequences used in this study were presented in Table 1.

**TABLE 1:** Primer sequences for RT-qPCR.

Gene (accession number)	Forward primers (from 5′ to 3′)	Reverse primers (from 5′ to 3′)
EIF2S3 (NM_001415.4)	GCAGTACACCTGACGAGTTT	CAACAAAGGAAACATGTCTGACTA
GTF3A (NM_002097.3)	CGAGTCGGTGTCGTCCTTGA	ACAAACAAATGGTCTCTCCCCC
HMGA1 (NM_001319078.2)	AGCGAAGTGCCAACACCTAA	CCATGGTGCCAACAAGAGGA
HSP90AB1 (NM_001271969.2)	GGTGGCCAACTCAGCTTTTG	GCCCCAGAATGGCTTACCTT
PABPC1 (NM_001438282.1)	AGCAAATGTTGGGTGAACGG	GCACAAGTTTCTTTTCATGGTCC
S100A11 (NM_005620.2)	AACCAGAAGGACCCTGGTGT	CAGAAGGGACAGCCTTGAGGAA
β-actin (NM_001101.5)	AGCGAGCATCCCCCAAAGTT	GGGCACGAAGGCTCATCATT

### Statistical analysis

Statistical analyses were performed using GraphPad Prism, v8.0 software. The differences between multiple groups were evaluated using one-way analysis of variance (ANOVA). Data were expressed as mean ± SEM, and the significance was defined as *P* < 0.05 (ns, not significant; *P < 0.05; **P < 0.01; ***P < 0.001).

## Results

### Transcriptome profile analysis of DEGs associated with the malignant potential and cancer risk of polyps

**FIGURE 2:**
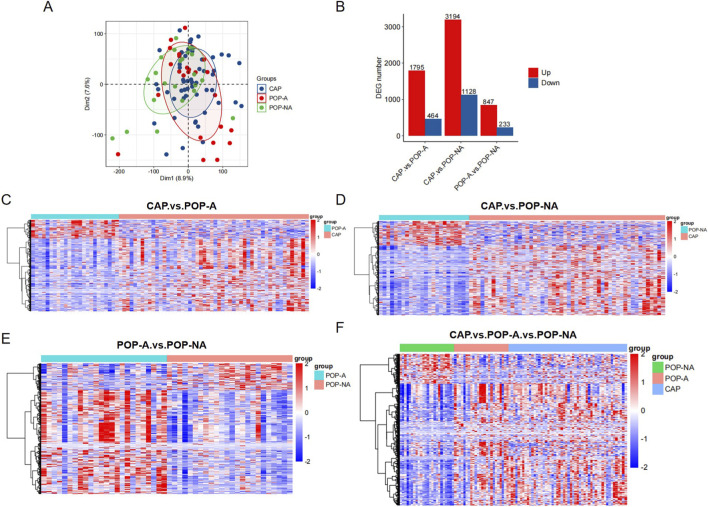
Transcriptome profile investigation of DEGs related to polyp malignant potential and CRC development risk. **(A)** Results of PCA of RNA-seq data. The figure shows the clustering of three polyp sample groups (POP-NA, POP-A, CAP). **(B)** Bar chart of DEGs. DEGs among the three sample groups were screened using the criteria of “FDR <0.05 and |log2FC| ≥ log2(1.5)”. **(C–F)** Heatmaps of DEGs expression. The figures visually display the hierarchical clustering results of DEGs across different groups, which helps identify expression trends related to polyp invasiveness and cancer association. FDR: false discovery rate; FD: fold change.

Identifying the molecular features of the transition from polyps to cancer is a crucial step in achieving early individualized management of polyps (Becker et al., 2022; Jass et al., 2006). We conducted an in-depth analysis of whole-genome sequencing data and RNA-sequencing from 100 patients by analyzing the dataset GSE209741, which includes cancer-free non-aggressive polyps (POP-NA) and aggressive polyps (POP-A), as well as cancer-adjacent polyps (CAP). First, the principal component analysis (PCA) was performed to identify the distinct clustering patterns among the different polyp groups (Figure 2A), the connection of the biological replicates revealed the quality of RNA-seq data is relatively reliable. We identified a set of differentially expressed genes (DEGs) among the three polyp groups (POP-NA, POP-A and CAP) using the screening parameters “FDR< 0.05 and |log2FC| ≥ log2(1.5)”. Results are presented as a bar chart (Figure 2B). Detailed gene information is provided in Supplementary Tables S2-S4. These genes may represent the key molecular differences between polyps in a cancer-related microenvironment and those with an aggressive phenotype that are still cancer-free. Furthermore, A heatmap was generated to visualize the expression patterns of the identified DEGs among different groups (Figures 2C–F). It was found that compared with either POP-A or POP-NA, genes in CAP were significantly upregulated, indicating that CAP is still distinctly different from POP. Genes in POP-A were also significantly upregulated compared with POP-NA, and many of these upregulated genes were simultaneously upregulated in CAP. Overall, these results provide valuable insights into the molecular differences among different types of polyps, which may contribute to the understanding of polyp malignancy and the development of potential diagnostic and prognostic markers.

### Enrichment analysis of DEGs among different groups

**FIGURE 3:**
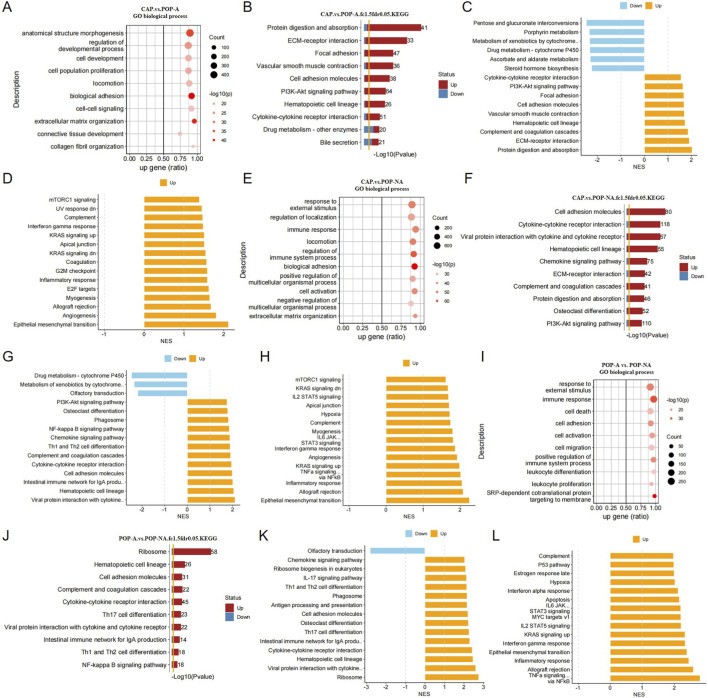
**(A–D)** CAP vs. POP-A. **(A)** GO biological process enrichment. Dot plot shows the top enriched BP terms. The x-axis represents the proportion of upregulated DEGs among all DEGs annotated to each term. Dot color indicates statistical significance (-log_10_(p value), darker shades denote more significant enrichment). Dot size reflects the number of DEGs in each term. **(B)** KEGG pathway enrichment. Bar plot displays the -log_10_(p value) for each pathway (longer bars indicate more significant enrichment). Bar fill color represents the ratio of upregulated to downregulated genes within the pathway (red: upregulated; blue: downregulated). **(C,D)** KEGG and Hallmark gene set enrichment analysis enrichment. The top 15 pathways ranked by p value are shown. Bars are colored by normalized enrichment score (NES): positive NES (red) indicates upregulation in CAP relative to POP-A; negative NES (blue) indicates downregulation. **(E–H)** CAP vs. POP-NA. **(E)** GO biological process enrichment. **(F)** KEGG pathway enrichment. **(G,H)** KEGG and Hallmark gene set enrichment analysis enrichment. **(I–L)** POP-A vs. POP-NA. **(I)** GO biological process enrichment. **(J)** KEGG pathway enrichment. **(K,L)** KEGG and Hallmark gene set enrichment analysis enrichment.

Next, enrichment analysis was performed on the DEGs among different groups to further evaluate the signaling pathways involved in the different groups. GO analysis indicated that compared with POP-A, the main biological processes enriched in CAP include structure morphogenesis, regulation of developmental process, cell development, cell population proliferation, biological adhesion and extracellular matrix organization (Figure 3A). KEGG analysis revealed that compared to POP-A, the adhesion and ECM-related signaling pathways were activated in CAP, while a suppression of metabolism associated signaling pathways such as pentose and glucuronate interconversions, porphyrin metabolism, metabolism of xenobiotics by cytochrome, Drug metabolism-cytochrome P450, ascorbate and aldarate metabolism and steroid hormone biosynthesis (Figures 3B,C). Analysis of the Hallmark gene set showed that the cell cycle, epithelial-mesenchymal transition, and inflammation response pathways exhibited varying degrees of activation, which further provides a molecular explanation for the malignancy and biological behavior of polyps (Figure 3D). By analyzing the differential genes between CAP and POP-NA, it was found that ECM and cytokine-related pathways were significantly activated, and these pathways are also associated with tumor progression (Figures 3E–H). Compared with POP-NA, POP-A is mainly enriched in immune regulation (such as Th1, Th2, Th17 cell differentiation, cytokine-cytokine receptor interaction, chemokine signaling pathway, interferon responses, *etc.*), cell survival, death and transformation (epithelial mesenchymal transition, hypoxia response, osteoclast differentiation, cell adhesion molecules), as well as signal transduction and regulation (KRAS signaling, IL6-JAK signaling, IL2-STAT5 signaling) (Figures 3I–L). Enrichment analysis of differential genes among CAP, POP-A, and POP-NA showed that pathways related to tumorigenesis are significantly activated during the progression of polyp malignant transformation.

### WGCNA and identification of key modules

**FIGURE 4:**
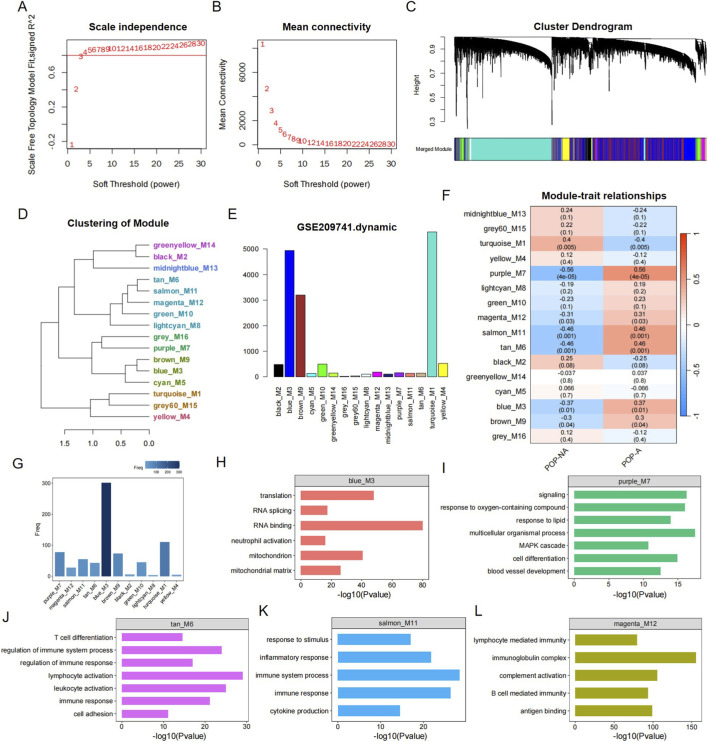
Weighted gene co-expression network analysis (WGCNA) of POP samples and identification of key modules associated with POP-A. **(A,B)** Selection of the soft-thresholding power β. Scale-free topology model fit index (*R* ^2^) as a function of soft-thresholding power β. The red dashed line indicates the threshold of *R* ^2^ = 0.8. β = 16 was selected as the lowest power achieving an *R* ^2^ of 0.879, satisfying the scale-free topology approximation criterion. **(C)** Gene dendrogram and module assignment. Hierarchical clustering tree (dendrogram) of genes based on topological overlap dissimilarity (1-TOM). Each leaf branch corresponds to an individual gene. The color row beneath the dendrogram represents the module assignment for each gene. 16 modules were identified. **(D)** Hierarchical clustering of module eigengenes (MEs) showing the dissimilarity between modules. **(E)** Bar plot showing the number of genes contained within each of the 16 modules. **(F)** Module–trait relationship heatmap. Pearson correlation between module eigengenes and phenotypic traits (POP-A vs. POP-NA). Each cell displays two values: the upper value is the Pearson correlation coefficient; the lower value in parentheses is the corresponding p-value. **(G)** Distribution of differentially expressed genes across modules. Histogram showing the number of differentially expressed genes (DEGs, POP-A vs. POP-NA) identified within each module. **(H–L)** Functional enrichment analysis of key modules. **(H)** M3, **(I)** M7, **(J)** M6, **(K)** M11, and **(L)** M12. Bar-plots display the top enriched GO biological process terms for each module.

Based on the previous analysis results, there is a significant difference between CAP samples and POP samples. Therefore, in the WGCNA analysis, we will only analyze the POP samples and evaluate these two groups (POP-A and POP-NA) through cluster analysis of their gene expression profiles. We used the soft threshold selection function and found that the soft threshold power value β = 16, the scale *R* ^2^ = 0.879, the RNA group had high average connectivity, and the connectivity between genes conformed to the scale-free network distribution (Figures 4A,B). Next, a topological overlap matrix (TOM) was constructed (Figure 4C), and a total of 16 modules were identified from the RNA co-expression network. We plotted the links between the indicated modules and the number of genes in each module (Figures 4D,E). We associated the modules with phenotypic attributes and screened out the modules with significant correlations (Figure 4F). According to the screening criteria (cor >0.3 and *p* value <0.05), the modules with a significant increase in POP-A were identified as M7, M12, M11, M6, M3, and M9. In addition, we found that among these modules, all except M9 showed an upward trend during the disease progression from POP to CAP (additional file 1: Supplementary Figure S1). Therefore, the relevant analysis of the M9 module was excluded in subsequent studies. Next, we conducted further analysis on the functions of the module genes. A histogram was used to show the number of differential genes in each module when comparing POP-A with POP-NA (Figure 4G). We found that M6, M11, and M12 are closely associated with biological processes such as leukocyte/lymphocyte activation, cytokine production, inflammatory response, immune response, T cell differentiation, immune system, and stress-related processes; Module M3 is related to transcriptional regulation and mitochondrial function regulation; Module M7 regulates cell differentiation, vascular development, signal transduction, and multicellular biological processes, and is involved in maintaining the growth, development, and homeostasis of organisms (Figures 4H–L).

### Single-cell RNA sequencing (scRNA-seq) data reveal cell heterogeneity during colorectal carcinogenesis

**FIGURE 5:**
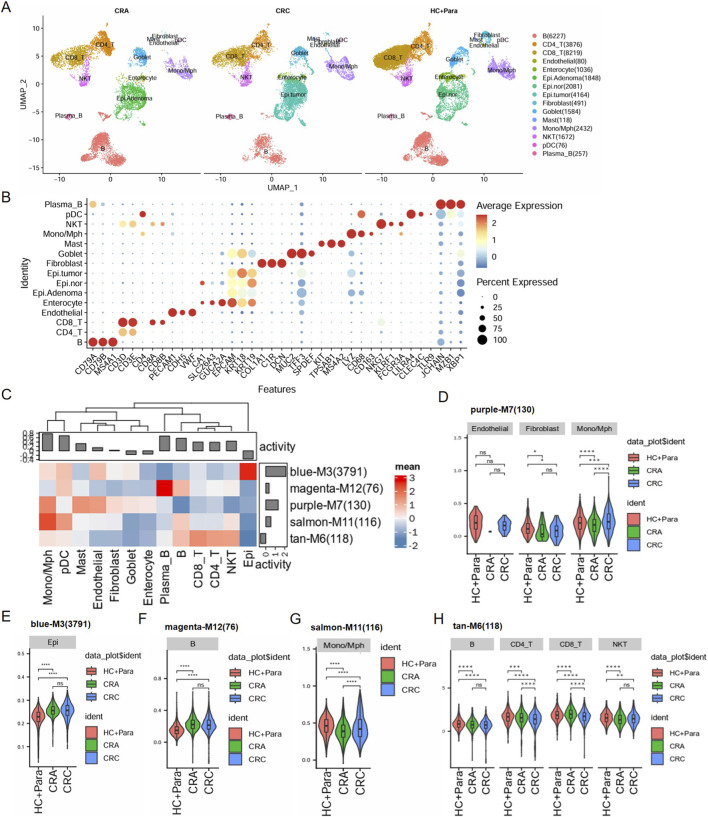
scRNA-seq analysis of cell heterogeneity during colorectal carcinogenesis. **(A)** UMAP visualization of cell clusters from para-cancer, adenomatous polyp, and cancer tissues, and cancer tissues. A total of 15 independent cell clusters were identified. **(B)** Bubble plot of canonical marker gene expression for each cell clusters. **(C)** Heatmap showing the expression scores of the key WGCNA module genes across all identified cell clusters. Higher expression levels were predominantly observed in specific cell clusters, suggesting cell type-specific enrichment of these co-expression modules. **(D–H)** Violinplot showing the expression trends of module genes in corresponding cells.

To dynamically understand the evolution of cell types and their molecular characteristics during colorectal carcinogenesis, we analyzed the transcriptomes of different populations in the GSE161277 dataset (Zheng et al., 2022). The core cells were classified into 15 independent cell clusters using the UMAP algorithm (additional file 2: Supplementary Figure S2), and the cell clusters of para-cancer, adenomatous polyp, and cancer tissues were visualized respectively to show the changes in molecular characteristics during colorectal cancer progression (Figure 5A). Marker genes were identified using the “SingleR” package, and the CellMarker database to annotate different clusters, the expression of important marker genes for each cell type was visualized by bubble plots (Figure 5B). Next, we verified the expression of key module genes across different cell clusters and observed the following: genes in module M3 are primarily localized in epithelial cells; those in module M12 are mainly expressed in B cells; module M7 genes are predominantly expressed in endothelial cells, fibroblast cells, and monocytes/macrophages cells; module M11 genes are mainly localized in monocytes/macrophages cells; and module M6 genes are primarily expressed in B cells, T cells and NKT cells (Figure 5C). We further analyzed the expression trends of module genes in the corresponding cells, and the results showed that: except for module M11, the genes of all other modules showed an upward expression trend with the increase in the malignancy of lesions when comparing para-cancer tissues with adenomatous polyps and cancer tissues (Figures 5D–H). These results not only clearly outline the evolution of molecular characteristics of cell types during the development of colorectal cancer, but also precisely identify the expression patterns of key module genes in different cell populations and their association with the malignancy of lesions.

### Intersection of genes within the module and differentially expressed genes in single cells—screening for key genes

**FIGURE 6:**
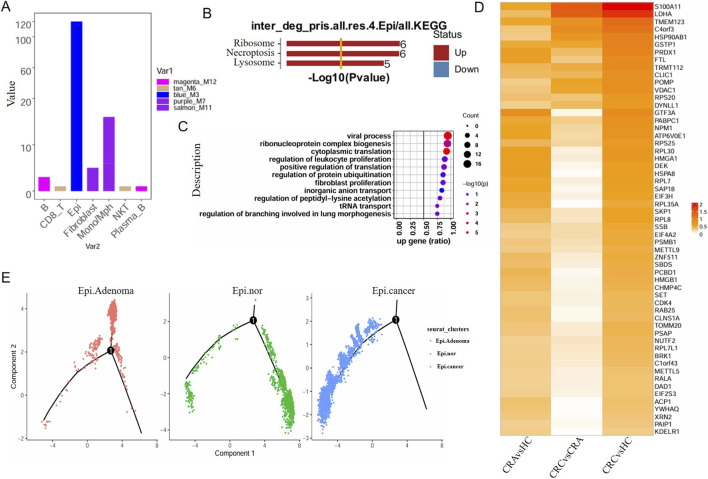
Screening of key genes by integrating WGCNA module genes and single-cell DEGs. **(A)** Statistical plot of shared genes between each WGCNA module and its corresponding cell cluster. **(B–C)** Functional enrichment analysis of epithelial cell-localized intersection genes. **(D)** Heatmap showing the log2FC value of 57 epithelial related genes between groups of samples. **(E)** Pseudotime analysis of epithelial cells during CRC progression reveals a linear evolutionary trajectory from normal epithelial cells to adenoma and subsequently to carcinoma.

Next, we conduct further analysis on the genes in the modules corresponding to specific cells. Our gene screening criteria are set as follows: First, in the comparison between adenomatous polyp and para-cancer, genes with |log2FC| ≥ 0.25 and P < 0.05 are screened out (i.e., genes whose expression is upregulated in adenoma relative to normal tissue); second, in the comparison between cancer tissues and adenomatous polyp, genes with |log2FC| ≥ 0 are further screened out (i.e., genes whose expression is higher in cancer tissue relative to adenoma tissue). Through the above two-step screening, the finally obtained gene set meets both the core conditions of “upregulated expression in adenoma compared with normal tissue” and “higher expression level in cancer tissue than in adenoma tissue”. The target genes obtained through the aforementioned screening were merged with the genes in each module of WGCNA. On this basis, the number of shared genes between each module and its corresponding paired cell cluster was statistically calculated (Figure 6A). We found that most of the screened genes are mainly localized in epithelial cells. Therefore, we conducted an enrichment analysis on the genes localized in epithelial cells, and the results showed that these genes are significantly enriched in the following biological processes: substance synthesis (translation, ribonucleoprotein complex biogenesis), functional regulation (ubiquitination, acetylation), homeostasis maintenance (lysosome, transport), life activities (proliferation, development), and pathological response (necroptosis, viral processes) (Figures 6B,C). The screening criteria were adjusted as follows: module genes with Kme >0.8 from the WGCNA analysis were merged with target genes from the single-cell dataset, resulting in a total of 57 genes localized in epithelial cells. Further heatmap analysis revealed that, among these genes, those significantly increased in adenoma tissues compared to para-cancer tissues were also significantly upregulated in the comparison of cancer tissues vs. para-cancer tissues (Figure 6D). In addition, pseudotime analysis was used to investigate the expression patterns of genes corresponding to epithelial cells from the precancerous state to the malignant state. It was found that most of these genes showed an upregulated trend during colorectal carcinogenesis (Figure 6E). Through multi-step screening and multi-dimensional analysis, these results clarify the expression characteristics and functional directions of key genes in epithelial cells, providing genetic-level support for the research on the mechanism of tumorigenesis and development.

### Feature selection of the malignant transformation markers in colorectal cancer based on machine learning algorithms

**FIGURE 7:**
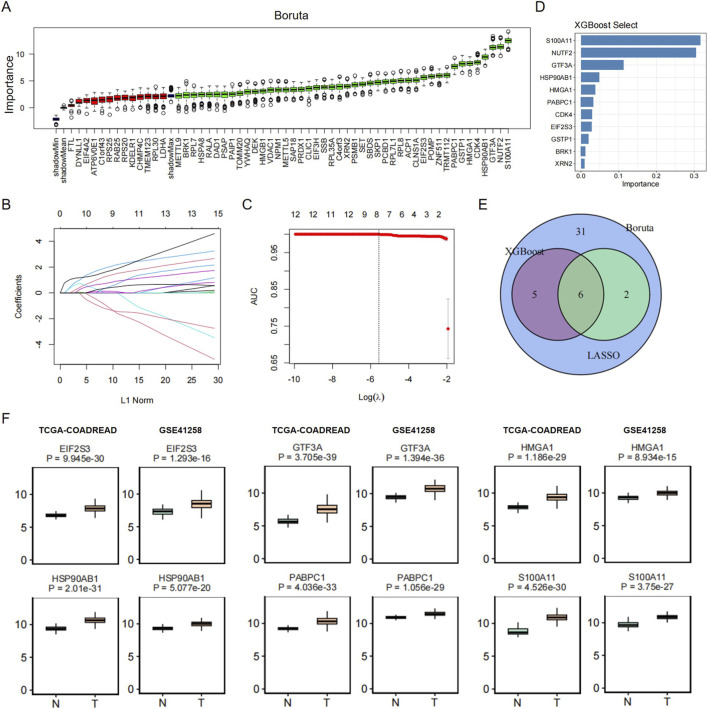
Screening of key feature genes based on machine learning algorithms. **(A)** The Boruta algorithm identifies genes with significant discriminative power. **(B,C)** LASSO regression screens for genes with non-zero coefficients. **(D)** XGBoost screens for key genes. **(E)** A Venn diagram was used to visualize and obtain the intersection of the screening results generated by the three algorithms. **(F)** Boxplots of the model genes were plotted based on the TCGA-COADREAD and GSE41258 datasets.

From the TCGA-COADREAD cohort retrieved from the TCGA database, eligible samples were randomly divided at a 7:3 ratio following screening, resulting in a training set of 443 cases (409 tumor samples, 34 normal samples) and an internal test set of 189 cases (175 tumor samples, 14 normal samples). Based on 57 pre-specified candidate genes, feature selection was performed in parallel on the training set using three algorithms: Boruta, LASSO regression, and XGBoost. The results indicated that the Boruta algorithm identified 44 genes with significant discriminative power, LASSO regression selected 8 genes with non-zero coefficients, and XGBoost yielded 11 key genes. By intersecting the results of the three algorithms, 6 genes (EIF2S3, GTF3A, HMGA1, HSP90AB1, PABPC1, S100A11) were ultimately determined as robust diagnostic feature genes for colorectal cancer, which constituted the final feature set (Figures 7A–E). This finding suggests that the feature genes validated by cross-algorithm verification exhibit good consistency and can serve as core targets for subsequent diagnostic model construction.

A ridge regression diagnostic model was constructed using the expression profiles of the 6 selected feature genes in the training set. Performance evaluation on the TCGA internal test set revealed that the model exhibited excellent efficacy in distinguishing primary tumor samples from normal solid tissue samples, with an accuracy of 99.32%, sensitivity of 99.27%, specificity of 100%, and precision of 100%. Further validation more intuitively confirmed that the model possessed strong diagnostic discriminative ability in the internal test set (accuracy: 98.41%, sensitivity: 98.29%, specificity: 100%, precision: 100%), enabling accurate differentiation between colorectal cancer tumor samples and normal tissue samples. To verify the robustness and generalizability of the model, the constructed ridge regression diagnostic model was applied to the independent external validation cohort GSE41258. This dataset comprises 240 samples (186 tumor samples, 54 normal samples) with a data structure consistent with that of the TCGA-COADREAD cohort, making it suitable for validating model generalizability. The results showed that the model still maintained excellent diagnostic performance in this cohort: accuracy of 91.25%, sensitivity of 99.46%, specificity of 62.96%, and precision of 90.24%. These results are highly consistent with those of the internal test set, indicating that the diagnostic model constructed in this study is not significantly affected by cohort heterogeneity and thus has good cross-cohort generalizability and potential for clinical application. Additionally, boxplots of the 6 model genes were generated based on the TCGA-COADREAD and GSE41258 datasets (Figure 7F).

### Expression profiling and survival analysis of malignant transformation markers in colorectal cancer

**FIGURE 8:**
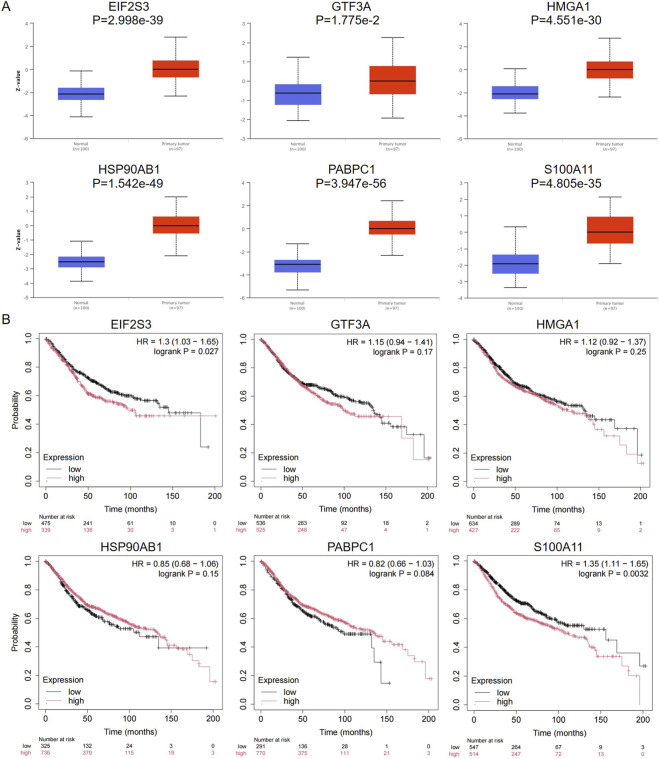
Expression and survival analysis of malignant transformation markers. **(A)** Protein expression profiles of these genes in normal and colorectal tumor tissues were inferred using the UALCAN online platform. **(B)** Kaplan–Meier survival curves illustrating the correlation between gene expression levels and overall survival of CRC patients. The x-axis represents time in months, and the y-axis represents the percentage of surviving patients. The red line represents patients with high gene expression, while the black line represents patients with low gene expression.

We employed the UALCAN website (https://ualcan.path.uab.edu/analysis.html) to examine protein expression levels, and observed a significant upregulation in the expression of these six genes in the tumor group compared with the normal group (Figure 8A). Subsequently, Kaplan-Meier survival analysis was conducted for these genes to evaluate their potential as prognostic biomarkers in CRC. After stratifying patients into low-expression and high-expression subgroups based on the expression levels of these genes, we found that notably, patients with high expression of EIF2S3 and S100A11 exhibited lower survival rates (*p* < 0.05), whereas no significant differences were observed for the remaining genes (Figure 8B). These findings suggest that EIF2S3 and S100A11 may serve as potential prognostic biomarkers for CRC.

### Validation of mRNA expression of key malignant transformation markers by qRT-PCR

**FIGURE 9:**
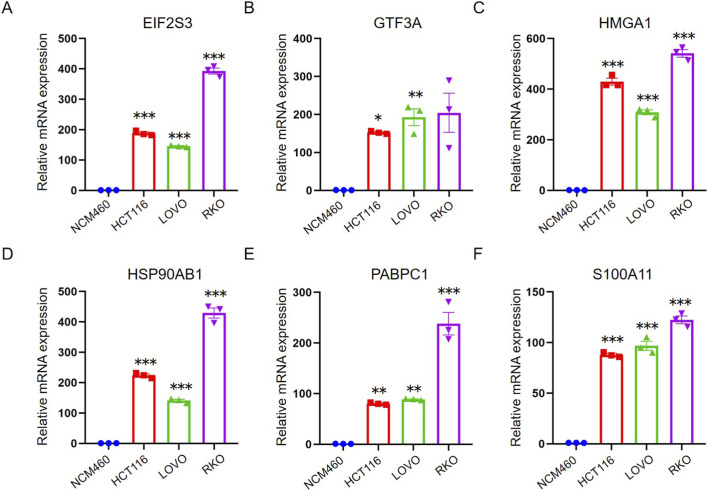
qRT-PCR validation of transcriptome expression levels of malignant transformation markers. **(A–F)** mRNA expression levels of EIF2S3, GTF3A, HMGA1, HSP90AB1, PABPC1, and S100A11 were measured by qRT-PCR in the indicated cell lines, including NCM460, HCT116, LOVO, and RKO. The abilities of each of the control cells were set as 1. *P < 0.05; **P < 0.01; ***P < 0.001.

Quantitative real-time polymerase chain reaction (qRT-PCR) assays were performed to determine the expression profiles of six key genes, utilizing total RNA extracted from the normal human colonic mucosal epithelial cell line NCM460 and three CRC cell lines, namely, HCT116, LOVO and RKO. The results demonstrated a marked and consistent upregulation of all six target genes in the CRC cell lines compared with their expression levels in the normal NCM460 cells (Figures 9A–F), thereby suggesting that these genes may play critical roles in the onset and progression of colorectal cancer.

## Discussion

Early prevention and management of colorectal cancer (CRC) represent the cornerstone of alleviating its global disease burden. Elucidating the molecular mechanisms governing the malignant transformation of colorectal precancerous lesions (i.e., colorectal polyps) into carcinoma, together with the identification of specific molecular biomarkers, serves as a core prerequisite for implementing early precision diagnosis and intervention. In this study, *via* integrative analysis of multi-dimensional datasets coupled with machine learning strategies, we systematically pinpointed six biomarkers mediating colorectal tumorigenesis (EIF2S3, GTF3A, HMGA1, HSP90AB1, PABPC1, S100A11), and further developed a ridge regression-based diagnostic model with robust performance. Collectively, our findings provide novel theoretical insights and potential therapeutic targets for the early diagnosis and mechanistic dissection of CRC.

Through multi-dimensional screening (module correlation analysis + single-cell localization validation + machine learning screening), this study ultimately identified six core genes, all of which exhibited high expression in colorectal tumor tissues and were closely associated with the core biological processes of tumorigenesis and progression. As a core subunit of the eukaryotic translation initiation factor 2 (eIF2) complex, EIF2S3 exerts its function in the early stage of protein synthesis by forming a ternary complex with GTP and initiator tRNA. Ben et al. confirmed that PROPER acts as a translational co-regulator in prostate cancer, and its pro-tumorigenic function is dependent on the regulation of the EIF2S3-YTHDF2/YBX3 axis (Ben et al., 2024). This is consistent with the finding of our study that high EIF2S3 expression correlates with the survival prognosis of colorectal cancer patients, suggesting that EIF2S3 may drive the malignant transformation of polyps into carcinoma by regulating translation initiation efficiency. As a transcription factor of RNA polymerase III, GTF3A participates in protein synthesis by regulating the transcription of non-coding RNAs such as tRNA, and its aberrant activation is closely related to malignant tumor proliferation (Wang et al., 2022; Luo et al., 2025). In esophageal squamous cell carcinoma, HMGA1 (high-mobility group protein A1) possesses dual properties as both a chromatin remodeling protein and a key immune checkpoint; it directly represses the transcription of STING by competitively binding to the transcription factor CREB against the coactivator CBP/p300 (Chen et al., 2025). As an important member of the heat shock protein 90 family, HSP90AB1 is highly expressed in advanced colorectal cancer tissues, and is closely correlated with tumor stage, lymph node metastasis and poor prognosis, thus serving as a predictive biomarker for the therapeutic response to HSP90 inhibitors (Schmitz et al., 2025). SUMOylation of PABPC1 constructs a core regulatory axis of “stress granule assembly–selective U-rich mRNA stabilization–mitophagy”, and acts as a key driver of cancer cell adaptation to stress (Huang C. et al., 2025). As a member of the S100 calcium-binding protein family, S100A11 has been confirmed to be associated with EMT, this protein is significantly upregulated in colorectal cancer, and knockdown of S100A11 can effectively inhibit the migration and invasion abilities of colorectal cancer cells (Huang Y. et al., 2025; Zhou et al., 2025). In addition, several studies have demonstrated that downregulated genes exhibit important diagnostic value in colorectal cancer. Asghari et al. found that CLCA1 and SELENBP1 were significantly downregulated in colorectal cancer tissues, while no significant differences were observed between normal and adenoma tissues, suggesting that these two genes could serve as potential biomarkers for the early diagnosis of colorectal cancer (Asghari Alashti et al., 2022). Niu et al. screened six genes that were markedly downregulated in colorectal cancer (PLCE1, PTGS1, AMT, ST8SIA1, ST3GAL5, and GBA2) and confirmed that their low expression was closely associated with tumor diagnosis and patient prognosis. Among them, downregulation of ST3GAL5 and GBA2 may also be involved in tumor progression by suppressing the malignant biological behaviors of colorectal cancer cells, further indicating that downregulated genes possess both important diagnostic value and functional significance (Niu et al., 2024).

The ridge regression diagnostic model constructed based on the core gene set exhibited excellent diagnostic efficacy in both the internal validation set and the external independent cohort (GSE41258), which fully verified the clinical diagnostic value of this core gene set. Currently used clinical methods for early diagnosis of colorectal cancer (such as fecal occult blood test and colonoscopy) have inherent limitations including insufficient sensitivity, strong invasiveness, and low patient compliance (Liu and Zhang, 2024; Rex et al., 2017). In contrast, the diagnostic model established based on gene expression signatures has unique advantages and application potential, such as high specificity and non-invasive detection capability, which is expected to effectively compensate for the shortcomings of existing diagnostic methods. In addition, qRT-PCR experiments confirmed that the transcription levels of these core genes were significantly elevated during the carcinogenesis of colorectal cancer. Further analysis using the UALCAN database verified that their protein expression levels were also significantly upregulated. Among them, the expression levels of EIF2S3 and S100A11 were closely correlated with the survival prognosis of patients, which provided a solid basis for the translation of these core genes from potential molecular markers to clinical therapeutic targets. For example, targeted inhibition of the translation initiation function of EIF2S3 or the activity of the signaling pathway mediated by S100A11 can block the malignant progression of colorectal polyps, thereby providing a novel strategy for the early intervention of CRC. Given that the biomarkers in this study were identified based on tissue transcriptome data from bioinformatics analysis, surgical or biopsy tissue specimens are recommended as the optimal starting material for clinical molecular detection. For future clinical translation, formalin-fixed paraffin-embedded (FFPE) tissues or fresh frozen tissues are both suitable for RNA-based validation and diagnostic applications.

The technical advantage of this study lies in the integration of a multi-dimensional research strategy. First, the WGCNA algorithm was applied to screen core module genes associated with disease progression from the GSE209741 dataset, which enhanced the pertinence of candidate genes. Second, combined with the single-cell transcriptome dataset, the expression localization of core genes in epithelial cells was clarified, which eliminated the interference from other cell types and improved the specificity of the results. Finally, three machine learning models, namely, the Boruta algorithm, LASSO regression and XGBoost, were jointly used to screen feature genes, which effectively reduced the noise interference of high-dimensional data and improved the reliability of core gene screening. This research paradigm of “bulk sequencing screening - single-cell sequencing localization - machine learning validation” provides a reference technical route for the screening of key genes in precancerous lesions.

This study has several limitations. (1) A major limitation of this study is the reliance on the phenotypic classification provided by the public GSE209741 dataset. The dataset contributors did not specify the detailed histopathological criteria used to classify “aggressive” and “non-aggressive” polyps. Since this grouping served as an important basis for study design and subsequent analyses, we cannot rule out phenotypic heterogeneity caused by unclear grouping definitions, which may to some extent affect the extrapolation and generalizability of the results. (2) The conclusions of this study are mainly based on bioinformatic analyses of public databases, with only preliminary validation at the cell line level and a lack of verification in clinical samples and *in vivo* functional experiments. Therefore, it is difficult to fully clarify the specific functions and molecular regulatory mechanisms of key genes during the malignant progression of colorectal polyps. Future studies should prioritize the validation of these six biomarkers in large-scale independent clinical sample cohorts (e.g., using FFPE samples and immunohistochemical assays) to confirm their expression characteristics at the protein level and clinical application value. (3) The single-cell transcriptomic analyses in this study focused only on epithelial cells and did not further explore the expression patterns and intercellular interactions of core genes in other cell types within the tumor microenvironment, which may overlook crucial multicellular regulatory networks involved in tumor progression. (4) The diagnostic model constructed in this study has only been retrospectively validated in existing public cohorts, and prospective multicenter and large-sample clinical studies have not yet been conducted. Therefore, its actual clinical application value still needs further confirmation. (5) This study did not perform systematic mining and analysis of downregulated genes. In future research, we will conduct basic validation and *in vitro* and *in vivo* functional experiments in larger clinical cohorts, further elucidate the molecular mechanisms of key genes, and continuously optimize the diagnostic model, so as to provide more reliable experimental evidence for early warning and precise intervention of malignant transformation of colorectal polyps.

## Conclusion

In summary, the six biomarkers identified in this study (EIF2S3, GTF3A, HMGA1, HSP90AB1, PABPC1, S100A11) are core regulatory factors for the malignant transformation of colorectal polyps. The diagnostic model constructed based on these genes exhibits promising clinical application potential. The findings of this study provide novel theoretical support for the early diagnosis, prognostic evaluation and therapeutic target development of colorectal cancer, and also offer new insights into the research on the molecular mechanisms of precancerous lesions.

## Data Availability

The datasets presented in this study can be found in online repositories. The names of the repository/repositories and accession number(s) can be found in the article/Supplementary Material.
