# Supplementary figures and images for: Bioinformatics combined with machine learning for the identification of malignant transformation markers in colorectal polyps
Source: Front Mol Biosci. 2026 Mar 24;13:1785464. doi: 10.3389/fmolb.2026.1785464 (PMC13053286; doi:10.3389/fmolb.2026.1785464)

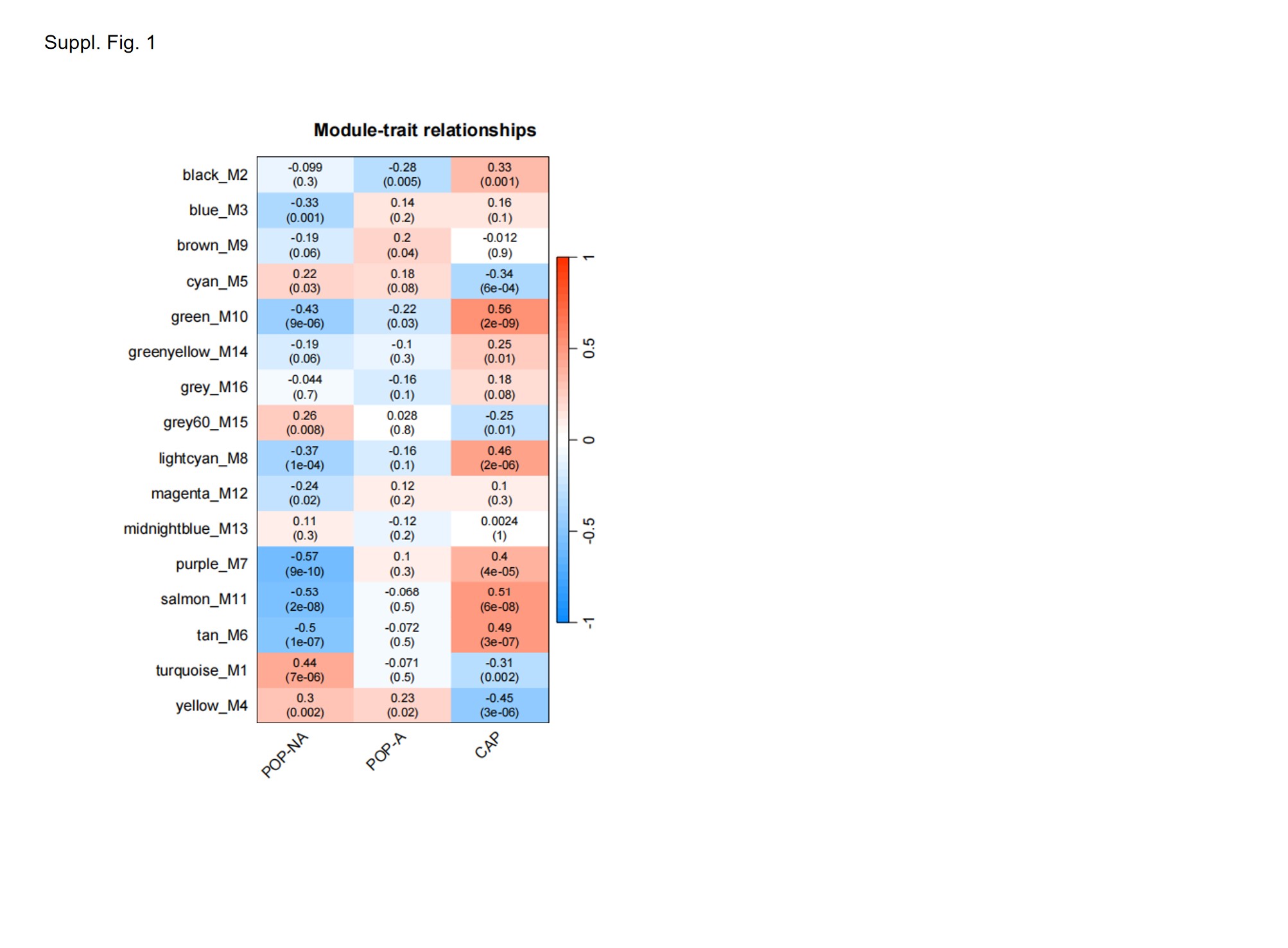

Supplement: Supplementary file 3 [file Image1.JPEG]

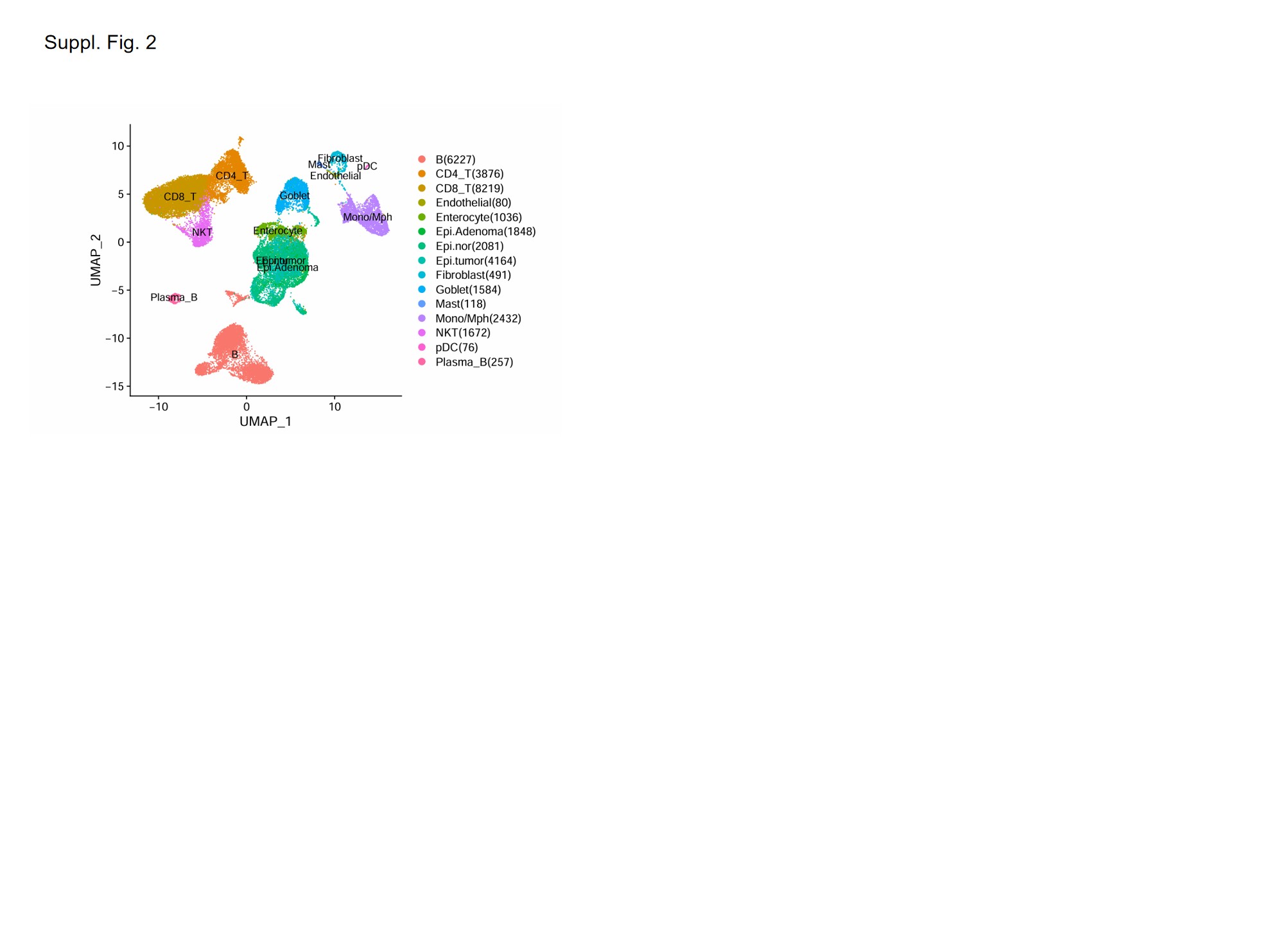

Supplement: Supplementary file 4 [file Image2.JPEG]
